# Crop Rotation and Straw Application Impact Microbial Communities in Italian and Philippine Soils and the Rhizosphere of *Zea mays*

**DOI:** 10.3389/fmicb.2018.01295

**Published:** 2018-06-15

**Authors:** Sarah A. Maarastawi, Katharina Frindte, Marius Linnartz, Claudia Knief

**Affiliations:** Institute of Crop Science and Resource Conservation (INRES), Molecular Biology of the Rhizosphere, University of Bonn, Bonn, Germany

**Keywords:** crop rotation, rice straw, rhizosphere, bacterial community, fungal community, *Zea mays*, paddy soil

## Abstract

Rice is one of the most important nourishments and its cultivation binds large agricultural areas in the world. Its cultivation leads to huge water consumption and high methane emissions. To diminish these problems, crop rotation between paddy rice and maize is introduced in Asia, but can lead to losses of carbon and water by the formation of desiccation cracks. To counteract these problems rice straw can be applied. We analyzed soil microbial responses to different crop rotation systems [rice–rice (RR), maize–maize (MM), maize–rice (MR)] and to rice straw application in the soil and rhizosphere of maize. *Zea mays* was grown in microcosms using soils from different field locations, each including different crop rotation regimes. The bacterial and fungal community composition was analyzed by 16S rRNA gene and ITS based amplicon sequencing in the bulk soil and rhizosphere. The microbiota was clearly different in soils from the different field locations (analysis of similarity, ANOSIM: R = 0.516 for the bacterial community; *R* = 0.817 for the fungal community). Within the field locations, crop rotation contributed differently to the variation in microbial community composition. Strong differences were observed in communities inhabiting soils under monosuccession (RR vs. MM) (ANOSIM: R = 0.923 for the bacterial and *R* = 0.714 for the fungal community), while the communities in soils undergoing MR crop rotation were more similar to those of the corresponding RR soils (ANOSIM: R = 0.111–0.175). The observed differences could be explained by altered oxygen availabilities in RR and MR soils, resulting in an enrichment of anaerobic bacteria in the soils, and the presence of the different crops, leading to the enrichment of host-plant specific microbial communities. The responses of the microbial communities to the application of rice straw in the microcosms were rather weak compared to the other factors. The taxa responding in bulk soil and rhizosphere were mostly distinct. In conclusion, this study revealed that the different agricultural management practices affect microbial community composition to different extent, not only in the bulk soil but also in the rhizosphere, and that the microbial responses in bulk soil and rhizosphere are distinct.

## Introduction

Rice is one of the most important staple foods worldwide and has an increasing demand because of the rising world population (Van Nguyen and Ferrero, 2006). Traditional rice cultivation causes major environmental problems as flooded rice fields represent a major source of atmospheric methane, a greenhouse gas that contributes 20–30% to global warming (Forster et al., 2007; Conrad, 2009). Moreover, rice cultivation under flooded conditions demands two to three times more water than the cultivation of other crops (Tuong et al., 2005). Especially in Asia, farmers need 50% of the freshwater to irrigate their paddy fields (Tuong et al., 2005). Because of these facts, farmers in tropical and subtropical Asia diversify their cropping systems by introducing a crop rotation system with paddy rice cultivation in the wet season and maize in the dry season (Weller et al., 2015). Maize already dominates the upland agricultural systems in the Philippines, because the requirement of water is much smaller and a simultaneous increasing demand of maize for poultry fattening and biofuel production has been reported (Weller et al., 2015).

The seasonal change between wet and dry conditions in the soil leads to the formation of desiccation cracks during maize cultivation, which cause loss of water, dissolved organic carbon and an increased release of the greenhouse gas N_2_O (He et al., 2015; Weller et al., 2015). To prevent crack formation and to stabilize the soil texture, rice straw can be incorporated into the soil at the end of the rice-cropping season. Additionally, surface-application of straw reduces evaporation and thus crack formation (Cabangon and Tuong, 2000). Moreover, straw application is known to improve the physical and biological conditions by preventing soil degradation and increasing soil organic carbon stocks and crop productivity (Chen et al., 2010; Liu et al., 2014).

The conversion of a rice monosuccession (RR) system into a maize–rice crop rotation system leads to altered conditions for soil microbial communities; especially the period of anoxic conditions in soil is considerably reduced. The archaeal community composition has been reported to show dramatic changes upon introduction of maize–rice crop rotation. A decrease of anaerobic methanogenic lineages and an increase of aerobic *Thaumarchaeota* was observed in the rotated soil during the rice growing season, whereas the bacterial community was only little affected (Breidenbach et al., 2015). In a different study, Lopes et al. (2014) reported responses of the bacterial community in a paddy soil undergoing crop rotation by introducing alfalfa as upland plant. Crop rotation may specifically affect microorganisms within microbial hot-spot regions of the soil such as the rhizosphere, as plant roots recruit their associated microbiota from the soil. This has not yet been studied in detail, especially for plants grown in paddy soils under upland conditions.

A modified crop rotation regime will lead to changes in soil carbon supply. All plants release a substantial amount of carbon into the soil via rhizodeposition (Bais et al., 2006; Badri and Vivanco, 2009). Due to the cultivation of maize in a rice cropping system, a different blend of carbon compounds will be released into the soil and a different, plant-host specific microbial community will develop in the rhizosphere (Berg and Smalla, 2009; Peiffer et al., 2013; Edwards et al., 2015). Over time, these changes may contribute to the development of an altered soil microbial community in maize–rice crop rotation systems compared to RR systems.

The application of rice straw provides a valuable carbon source for soil microorganisms, and rice straw degradation has been studied in paddy soils under oxic as well as anoxic conditions. Chen et al. (2010) and Conrad et al. (2012) showed that the addition of straw leads to changes in the bacterial and archaeal community composition in paddy soils. An effect was also seen in the rice rhizosphere (Shrestha et al., 2011). Different bacterial and fungal taxa were identified as part of the straw degrading community (Rui et al., 2009; Lee et al., 2011; Shrestha et al., 2011; Murase et al., 2012). However, the impact of rice straw addition on microbial communities in paddy soils was mostly studied in soils of RR systems, while information about the short-term effects of rice straw in crop rotation systems is scarce, where the applied rice straw is mostly degraded under oxic conditions during the period of upland cropping.

The objective of this study was to investigate how agricultural management practices affect soil microbial communities, focussing on the longer-term effects of crop rotation and the short-term responses of straw application. Responses of bacterial and fungal communities were analyzed in bulk and rhizosphere soil. We hypothesized that (1) long-term monosuccession of rice and maize leads to the development of clearly distinct microbial communities in these soils, while the implementation of a maize–rice crop rotation will cause moderate shifts, leading to communities with intermediate appearance. Crop rotation will induce specific microbial responses in the bulk soil and the rhizosphere of the cultivated crop. (2) The application of rice straw will change microbial community diversity and composition. We expect to see stronger responses in the bulk soil microbial communities than in the rhizosphere microbiota, as the latter is expected to profit predominantly from plant root derived carbon. To address these points, we analyzed the composition of bacterial and fungal communities in soils subjected to different crop rotation regimes, i.e., we compared maize monosuccession (MM) or maize–rice crop rotation (MR) to RR. Soils were collected from different field sites and used in microcosm experiments, in which maize was cultivated in the presence or absence of rice straw. Bulk soil samples as well as rhizosphere samples were collected at different time points during the maize growth period.

## Materials and Methods

### Sampling Sites

Field locations for soil sample collection were at the International Rice Research Institute (IRRI) in Los Banos, Philippines (14°11′N, 121°15′E), in Tarlac, Philippines (15°32′N, 120°37′E) and Zeme, Pavia, Italy (45°11′N, 8°40′E). From each study site, we took soil from two neighboring fields, which were under different crop rotation regimes for different periods of time (**Table 1**). Each site included a field under RR, to which the respective alternative cropping regimes (MR or MM) were compared. Soils with different periods of maize rice crop rotation were taken from IRRI (MR crop rotation since 4 years) and Tarlac (MR crop rotation since 20 years). Soil under MM (>30 years) from Italy was included to compare the impact of MR crop rotation to differences developing under long-term monosuccession regimes. At the Philippine sites, soil samples were taken at the end of the wet season after the rice cultivation period and thus before rice and maize planting. Likewise, the Italian soils were collected in spring before rice/maize planting. All samples were taken from drained soils after plowing. Upon collection, the soil was immediately air-dried and homogenized before the start of the experiment. Basic soil properties including soil type, maximum water holding capacity, pH, nitrogen and carbon content, and the C:N ratio were determined using standard methods (Supplementary Table 1).

**Table 1:** Experimental setup of the microcosm experiment.

Field location	Crop rotation	Experimental site for microcosm experiment	Maize variety	Time (sample collection time points, days after sowing)
Italy	Rice (RR) Since >30 years	Bonn, Germany	NC358	0, 8, 15, 29, 43, 85
Italy	Maize (MM) Since >30 years	Bonn, Germany	NC358	0, 8, 15, 29, 43, 85
IRRI, Philippines	Rice (RR) Since >50 years	IRRI, Philippines	Pioneer 30T80	0, 15, 43
IRRI, Philippines	Maize–Rice (MR) Since 4 years	IRRI, Philippines	Pioneer 30T80	0, 15, 43
Tarlac, Philippines	Rice (RR) Since 1 year	IRRI, Philippines	Pioneer 30T80	0, 15, 43
Tarlac, Philippines	Maize–Rice (MR) Since >20 years	IRRI, Philippines	Pioneer 30T80	0, 15, 43


### Setup of the Microcosm Experiment

For the microcosm experiments, the soil was moistened, half of it mixed with chopped rice straw (2–5 cm pieces; 6 kg straw m^-3^), filled into plastic pots (1.2 L pots for sample collection form young plants, 7 L pots for plants older than 21 days), and maize seeds were sown. For bulk soil sampling, pots remained unplanted. The experimental setup included four replicates for all treatments (i.e., for maize with straw, maize without straw, bulk soil with straw, and bulk soil without straw) and every time point. The first rhizosphere samples were taken 8 days after sowing, while bulk soil sampling started at the day of sowing; further sampling was performed as listed in **Table 1**. The pots were watered every day and received basal fertilization of 50 kg P_2_O_5_ ha^-1^ and 30 kg K_2_O ha^-1^ at seeding. Nitrogen was applied in three split applications with 30 kg urea ha^-1^ basal at seeding and 50 kg urea ha^-1^ at 29 and 50 days after seeding. The greenhouse experiments with the soils from Italy were conducted in Bonn (Germany), while the experiments with soils from Tarlac and IRRI were conducted at IRRI, Philippines. For the Italian soils, sampling was performed with high temporal resolution until day 85 to evaluate time-dependent responses in detail, while a lower temporal resolution was chosen for the Philippine samples (**Table 1**).

### Rhizosphere and Bulk Soil Sample Collection

About 10 g of bulk soil were taken with a sterile spatula from unplanted pots after mixing the soil in the pot and immediately frozen at -20°C. For rhizosphere sampling, plants were removed from the pots and hand shaken to remove large soil aggregates and loosely adhering soil. The soil remaining attached on the roots was considered to be rhizosphere soil and was collected using a modified protocol of Lundberg et al. (2012). Roots with associated rhizosphere soil were placed into a sterile 50 mL tube and submerged with 25 mL 1× phosphate buffered saline (PBS; 1.36 M NaCl, 100 mM Na_2_HPO_4_, 20 mM KCl, 17 mM KH_2_PO_4_, 0.02% Silwet L-77, pH 7.4). Larger samples, collected from plants older than 28 days, were transferred into a sterile 720 mL glass and filled with 300 mL 1× PBS. Thirty or fifty grams (for the larger samples) of sterile glass beads (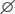 4 mm) were added and the samples shaken at 420 rpm for 20 min. The resulting turbid solution was filtered through a 500 μm nylon mesh into new 50 mL tubes to separate soil and roots. The filtrate was centrifuged for 20 min at 3200 × g. The supernatant was removed and the pellet stored at -20°C until further processing.

### Nucleic Acid Extraction and Amplicon Sequencing

Soil DNA extraction was performed using the NucleoSpin Soil Kit (Macherey Nagel, Düren, Germany) following the manufacturer’s instructions with the following modifications. Microbial cells in 0.3 g of soil were mechanically disrupted by beat beating (TissueLyser, Qiagen, Germany) in the presence of SL1 buffer solution and enhancer solution. The final resuspension of DNA was done in 30 μL PCR-grade water.

16S rRNA genes were amplified using the primer set 515F-806R, targeting a 291 bp product of the V4–V5 region of the 16S rRNA gene from *Bacteria* and *Archaea* (Bates et al., 2011). The fungal ITS1 region was amplified using the primer set ITS1F-ITS2 (White et al., 1990; Gardes and Bruns, 1993), resulting in a 180 bp product. We used a two-step PCR approach in which conventional PCR primers without barcodes were applied to amplify the target region during 30 cycles in the first step. In the second step, the obtained amplicons served as template in a 5 cycle PCR using sample-specific barcode primers. The forward primer included an 8-bp barcode plus a 3–5 bp stagger sequence to increase sequence variability for the Illumina platform. PCR reactions were carried out in technical triplicates and pooled for sequencing. Each replicate 25-μL assay contained 1× Herculase II reaction buffer, 0.25 U Herculase II Fusion DNA Polymerase (Agilent Technologies, Santa Clara, CA, United States), 0.25 mM dNTPs, 0.25 μM of forward and reverse primer, 1 mM MgCl_2_, 0.8 mg mL^-1^ BSA and 1 μL template DNA. The thermal cycling protocol consisted of an initial denaturation step at 95°C for 2 min, followed by repeated cycles of denaturation at 95°C for 20 s, annealing at 52°C for the 16S rRNA gene and 50°C for the ITS1 region for 20 s, elongation at 72°C for 20 s and a final elongation step for 3 min.

The PCR products were quantified using the QuantiFluor dsDNA System (Promega, Madison, WI, United States) on an Infinite 200 Pro plate reader (Tecan, Männedorf, Switzerland) at 490 nm excitation and 530 nm emission wavelength. Afterwards, PCR products were pooled at equimolar concentrations. Pooled PCR products were cleaned using the CleanPCR magnetic bead system (CleanNA; Alphen aan den Rijn, Netherlands) according to manufacturer’s instructions. Library preparation and sequencing on an Illumina HiSeq system generated paired-end reads (2 × 250 bp) and was performed by the Max Planck-Genome-centre Cologne. Read files obtained after the quality filtering step were submitted to the European Nucleotide Archive (ENA) database under the project accession number PRJEB23682.

### DNA Sequence Analysis

Sequence data were assembled with the USEARCH paired-read assembler (Edgar and Flyvbjerg, 2015) to create consensus sequences with a consensus of at least 90% and a quality score of *Q* = 2. Sequences were trimmed to remove the reverse primer and sequences <200 bp were removed (*cutadapt*) (Martin, 2011). Reverse complementary sequences were identified and turned (*fastx_reverse_complement*). The sequences were demultiplexed according to their barcode sequences using an own written perl script. The forward primer was removed after demultiplexing, because barcode sequences are located in front of the forward primer.

Quality filtering and dereplication were conducted using USEARCH v9 (Edgar, 2013) (USEARCH parameter: remove reads with expected number of base call errors exceeding *p* = 0.01 and *Q* = 20). The sequences were binned into operational taxonomic units (OTUs) at a threshold of 97% similarity (corresponding to genus level resolution) using the UPARSE algorithm (Edgar, 2013). This command included chimera filtering. An additional identification of chimeric sequences was done using the uchime2 algorithm (Edgar, unpublished) on USEARCH 9.0 against a reference database. 16S rRNA gene based OTUs were annotated based on representative sequences according to the RDP 16S rRNA training set v16 (Maidak et al., 2000; Edgar, unpublished), while ITS OTUs were taxonomically identified according to the UNITE ITS database (version 7.1; Abarenkov et al., 2010). For taxonomy prediction, a cut-off value of 0.8 was chosen. Finally, 16S rRNA gene sequences identified as chloroplasts were removed from the 16S rRNA gene sequence dataset (0.5% of the sequences).

### Statistical Analysis

Statistical analyses were conducted in STAMP (Parks and Beiko, 2010) and in R using the packages Vegan (Oksanen et al., 2016) and Phyloseq (McMurdie and Holmes, 2013). For all OTU based analyses, the original OTU table was filtered to contain only sequences that were taxonomically classified as *Fungi* or *Bacteria* and *Archaea* and OTUs represented by a maximum of only two reads in one or more samples were discarded. Estimation of Alpha-diversity was based on an evenly rarefied OTU table and included calculation of the observed richness via Chao1. To test for significant differences in bacterial and fungal Alpha-diversity between groups of samples, non-parametric Kruskal–Wallis tests were performed, as Shapiro–Wilk test revealed non-normal data distribution.

The structure of the microbial communities was evaluated at high taxonomic resolution (97% sequence identity) and ordinated in non-metric multidimensional scaling (NMDS) plots based on Bray–Curtis dissimilarity matrices. For NMDS ordination, the OTU tables were pre-processed, so that sparse OTUs were removed. The sparsity threshold was 0.5, meaning that an OTU not found in at least 50% of the samples was removed as statistically uninformative. To test for significant differences between groups of samples, an analysis of similarity (ANOSIM) was performed in Vegan with 999 permutations based on Bray–Curtis distances between samples. In case of multiple comparisons, *P*-values were Bonferroni-Holm corrected. NMDS and ANOSIM were performed on successively reduced datasets, beginning with an overall analysis (including all locations, compartments, time points, and straw treatments). This was followed by the analysis of subsampled datasets to assess the impact of crop rotation and straw treatment in more detail. Crop rotation was evaluated (i) within each field location (including compartments, time points, and straw treatments), (ii) within each compartment (including time points and straw treatments), (iii) at different time points (including straw treatments). For straw treatment, the data were completely dissected. This procedure followed the succession from the most significant to the least significant impact factor, enabling to evaluate the effect of each treatment more specifically and to exclude effects of co-variants.

The impact of different treatments on individual taxa was analyzed using the program STAMP (Parks and Beiko, 2010). First, genera were identified that responded to crop rotation within each field location. The datasets were then further subsampled by compartment to compare crop rotation responsive taxa between bulk soil and rhizosphere. To identify genera that were significantly impacted by straw addition, the datasets were subsampled according to field location, crop rotation, and compartment. Significant differences between groups of samples were tested with Kruskal–Wallis *H* test and multiple comparison corrections were done with Benjamini-Hochberg FDR.

## Results

### Identification of Major Factors Affecting Diversity and Composition of Bacterial and Fungal Communities

Bacterial and fungal communities were analyzed in soil and rhizosphere samples by 16S rRNA gene and ITS1 amplicon sequencing, respectively. After pre-processing and quality filtering, about 16,500 reads per sample of 16S rRNA gene sequences and 13,900 reads per sample of the ITS1 region remained. In a first instance, the data of all samples were combined to identify major factors that influenced microbial diversity and community composition. The effects of crop rotation, straw treatment, field location, and compartment as well as variation due to incubation time were evaluated. A comparison of OTU richness and Chao 1 diversity indices revealed that all factors had a significant influence on bacterial and fungal diversity (Supplementary Table 2). The diversity was most strongly influenced by crop rotation and least by straw application (in case of bacteria) and compartment (in case of fungi).

**FIGURE 1:**
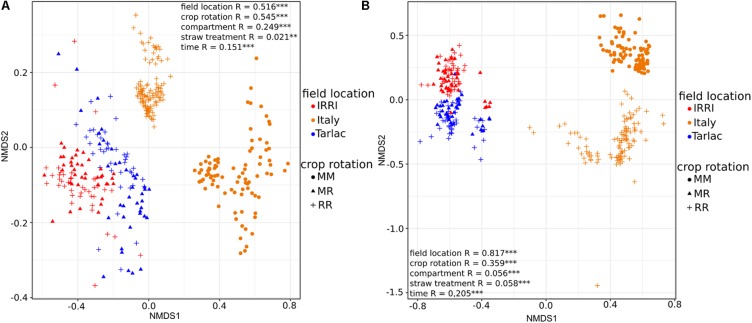
Ordination plots showing the influence of field location and crop rotation on bacterial **(A)** and fungal **(B)** community composition. Non-metric multidimensional scaling (NMDS) plots based on Bray–Curtis dissimilarities were calculated from relative OTU abundances. Results of analysis of similarity (ANOSIM) are shown with ^∗^*P* < 0.05, ^∗∗^*P* < 0.01, ^∗∗∗^*P* < 0.001 for all grouping factors.

In all soils the bacterial communities were dominated by the phyla *Acidobacteria*, *Chloroflexi*, and *Proteobacteria* (classes *Alphaproteobacteria*, *Betaproteobacteria*, *Gammaproteobacteria)*, while fungal communities consisted mainly of members of the classes *Sordariomycetes*, *Dothideomycetes*, *Eurotiomycetes*, and *Agaricomycetes* (Supplementary Figure 1). Differences in the composition of the microbial communities between all samples based on NMDS plots in combination with ANOSIM revealed that field location and crop rotation were the most relevant factors explaining dissimilarities between samples (**Figure 1**). ANOSIM showed that field location affected in particular the fungal communities, as evident from the high *R*-value of 0.817 (*P* < 0.001), while variation in bacterial communities due to field location resulted in an intermediate *R*-value of 0.516 (*P* < 0.001). The effect of crop rotation on bacterial communities was also intermediate (*R* = 0.545; *P* < 0.001), while it was lower for fungal communities (*R* = 0.359; *P* < 0.001). The factors compartment, time and straw had a weaker effect on the overall microbial community composition. These findings were confirmed by a cluster analysis, performed at class level resolution (Supplementary Figure 2). Because of the strong differences due to field location, the samples from each location were analyzed separately to assess the impact of crop rotation and straw application in more detail.

### Impact of Crop Rotation on Microbial Diversity and Community Composition Within Field Locations

**FIGURE 2:**
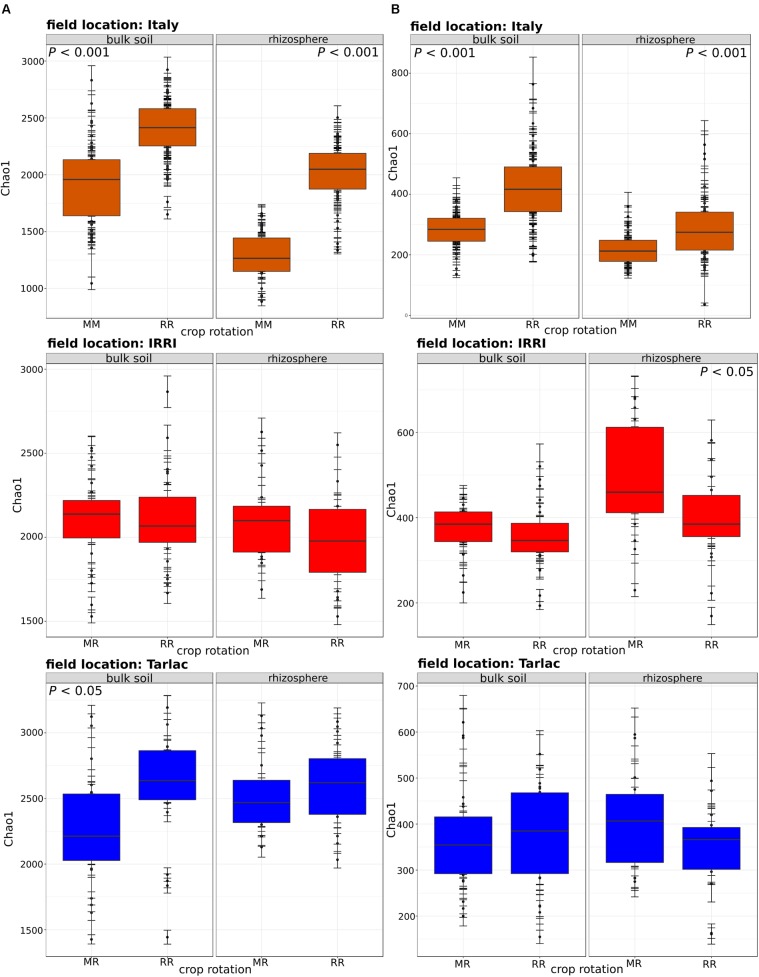
Box plots showing the influence of crop rotation on bacterial (column **A**) and fungal alpha diversity (column **B**) within each field location and compartment. Each box plot shows alpha diversity based on the Chao1 diversity index and includes all samples treated with or without straw and collected at different time points. The median as well as the 25^th^ and 75^th^ percentile of the samples is presented, individual data points outside of this range are given as mean ± standard deviation of four replicate samples. Significant differences due to crop rotations within one compartment (bulk soil or rhizosphere) are noted.

Strongest differences in diversity in response to crop rotation were observed in the Italian soil samples (**Figure 2**), where the diversity of bacteria and fungi was 1.3- to 1.5-fold higher in RR soil than in MM soil (*P* < 0.001). This was observed in the bulk soil as well as in the rhizosphere. In IRRI and Tarlac soils, crop rotation affected microbial diversity less consistently. The fungal diversity was modulated by crop rotation in the rhizosphere of IRRI soil and the bacterial diversity in the bulk soil of Tarlac (*P* < 0.05).

**FIGURE 3:**
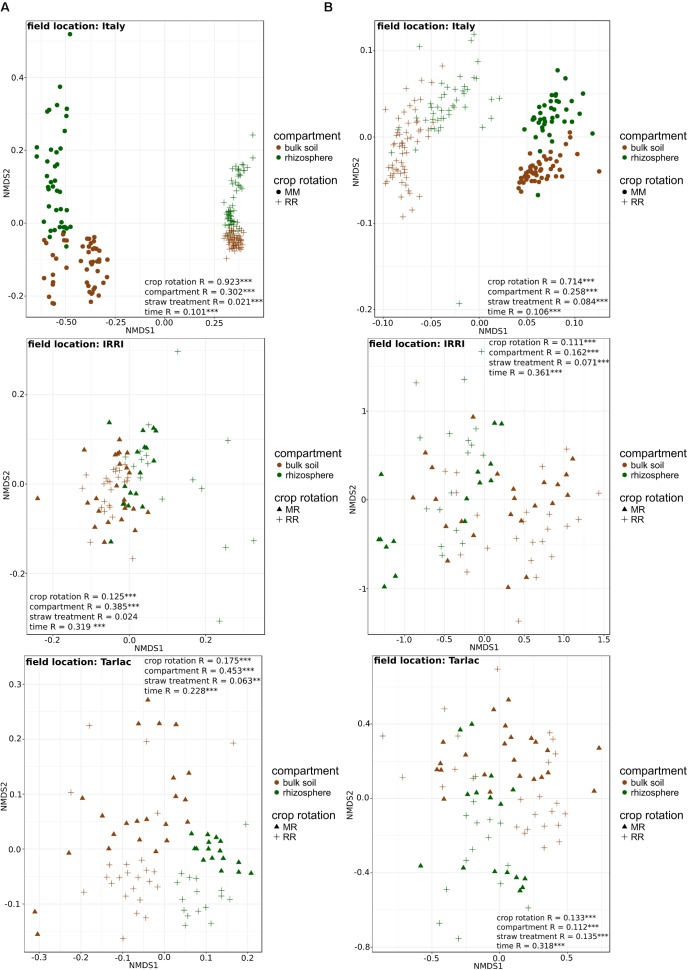
Non-metric multidimensional scaling (NMDS) plots of 16S rRNA (column **A**) and ITS1 (column **B**) community composition in soils from different field locations. NMDS plots based on Bray–Curtis similarities were calculated from relative OTU abundances. ANOSIM was applied to test for differences in community composition due to compartment, crop rotation, time and straw treatment. *R*-values are shown with ^∗^*P* < 0.05, ^∗∗^*P* < 0.01, ^∗∗∗^*P* < 0.001.

Field location specific NMDS ordinations along with ANOSIM (**Figure 3**) revealed that crop rotation had a very strong impact on the bacterial (*R* = 0.923; *P* < 0.001) and fungal (*R* = 0.714; *P* < 0.001) community composition in the Italian soils, where rice and maize were grown in monosuccession for >30 years, respectively. A further clear structuring of the communities in these soils was evident according to compartment (*R* = 0.302 for bacteria, *R* = 0.258 for fungi; both *P* < 0.001), while the factors time and straw application explained less variation. In the Philippine soils, which were managed under RR or MR, crop rotation explained less of the variation in microbial community composition (*R*-values between 0.111 and 0.175) and was in most cases less important compared to the impact of compartment and time (*R*-values between 0.112 and 0.361). As the impact of crop rotation was covered by compartment and time in these soils, it was evaluated more specifically by ANOSIM within each compartment and at each time point (Supplementary Table 3). This revealed a significant response of the microbial community to crop rotation in all individual cases. The response remained strongest for the soils from Italy and was of equal strength in the soils from IRRI and Tarlac. Moreover, crop rotation had a stronger effect on the bacterial than the fungal community (*P* < 0.005). Responses in the bulk soil and rhizosphere were of comparable strength, and a trend over time was not evident.

**FIGURE 4:**
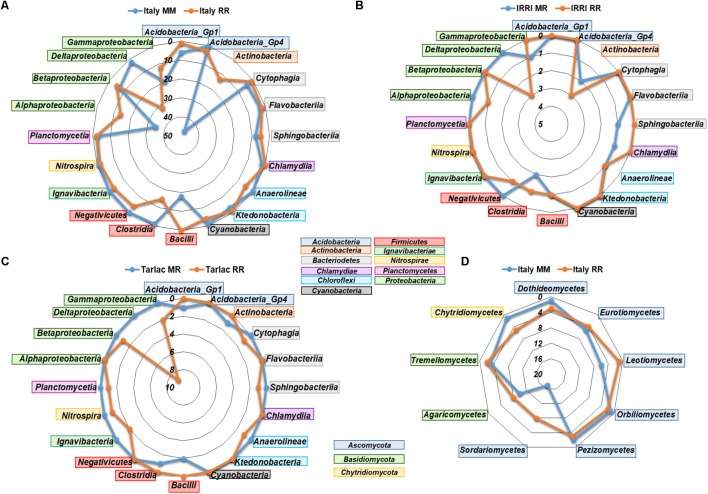
Radar charts showing the number of bacterial **(A–C)** and fungal **(D)** genera in different classes that were identified as significantly enriched by crop rotation in the different field locations (Italy, IRRI, Tarlac) based on STAMP analysis. Displayed are classes for which at least three different genera were identified as specifically enriched in one or the other soil. Fungal responses to crop rotation at IRRI and Tarlac were weak and are therefore not included in the figure.

We performed analyses in STAMP to identify bacterial and fungal genera that responded to crop rotation within each field location. In agreement with the clear impact of crop rotation at the Italian site, the highest number of responsive genera (361 bacterial and 94 fungal) was observed here (listed in Supplementary Tables 6A,B), while the numbers were much lower in soils from IRRI and Tarlac (25 and 41 bacterial genera, 4 and 5 fungal genera). More than half of the genera that were identified as responsive in a Philippine soil showed a similar response in soils from Italy. Most responses were observed within the phyla *Actinobacteria*, *Acidobacteria, Firmicutes*, and *Proteobacteria*. Genera of the classes *Actinobacteria*, *Alphaproteobacteria*, *Gammaproteobacteria*, and *Bacilli* were predominantly enriched in the Italian MM soil (**Figure 4**). Likewise, genera of *Actinobacteria*, *Bacilli*, and *Gammaproteobacteria* were enriched in the Philippine MR soils. In contrast, genera of *Deltaproteobacteria*, including sulfate and iron reducers, were consistently enriched in all RR soils. Moreover, diverse genera of *Acidobacteria*, *Actinobacteria*, *Chloroflexi*, *Firmicutes* and the class *Alphaproteobacteria* were specifically enriched in some of the RR soils. In the fungal communities, most differences were observed within the phyla *Ascomycota* and *Basidiomycota.* Especially genera of the class *Sordariomycetes* and *Agaricomycetes* were enriched in the Italian RR or MM soil (**Figure 4**).

Because compartment-specific responses to crop rotation were of particular interest, an additional analysis in STAMP was done with datasets separated further by compartment. Overall, the analysis resulted in a comparable number of responsive genera in MM, MR, and RR soils at the respective field locations (listed in Supplementary Tables 6C,D). Thus, the further dissection of the datasets according to compartment did not result in the identification of a higher number of responsive taxa in the Philippine soils, where compartment masked the effect of crop rotation to some extent. As before, differences were most evident in soils from Italy and more differences were observed for bacterial than fungal communities. Focussing on compartment-specific responses to crop rotation in the Italian soils, the analysis revealed a higher number of responsive bacterial genera in the bulk soil (165 specific for MM and 176 for RR) than in the rhizosphere (143 specific for MM and 126 for RR). The percentage of bacterial genera that responded in both compartments was high, with 45 and 60% for MM and RR soils, respectively (Supplementary Figure 3). The same trends were observed in most Philippine soils. Fungal communities showed less overlap (maximum 25%) between compartments. Among the genera that were enriched in the Italian MM soils, members of *Bacilli* and *Gammaproteobacteria* were more specifically found in the bulk soil, while *Betaproteobacteria* were more specifically responding to crop rotation in the rhizosphere (Supplementary Figure 4). In the Italian RR soil, genera of *Actinobacteria*, *Clostridia*, *Alphaproteobacteria*, and *Deltaproteobacteria* were more responsive to crop rotation in the bulk soil, likewise as the fungal genera of the class *Sordariomycetes*.

### Impact of Straw Application on Microbial Communities

**FIGURE 5:**
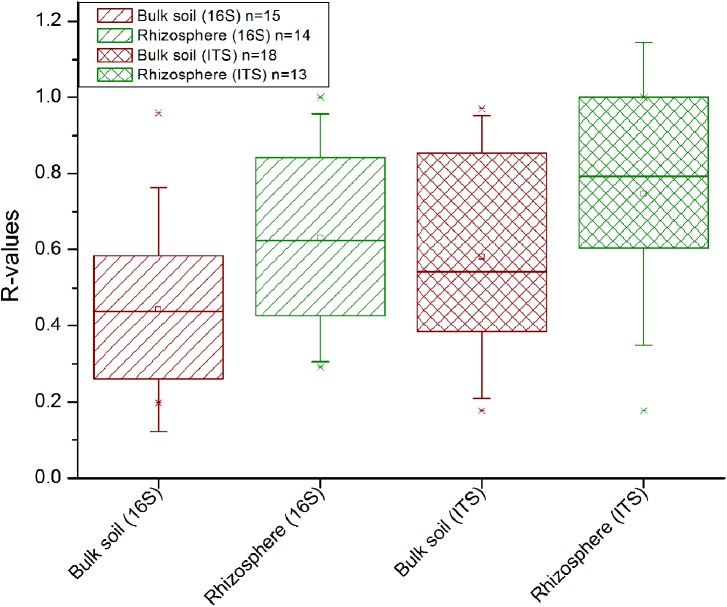
Influence of straw treatment on the bacterial (16S) and fungal (ITS1) community composition in the rhizosphere and bulk soil. Data from each field, each compartment and of every time point were analyzed separately by ANOSIM. All significant *R*-values from this analysis (as shown in Supplementary Table 4) are included in this plot. Between 62 and 78% of the *R*-values were significant (*P* < 0.05) per group displayed.

In comparison to the other factors, straw application had the weakest impact on the bacterial and fungal diversity (Supplementary Table 2) and community composition (**Figure 3**). ANOSIM revealed that a straw effect was most evident in the fungal community in Tarlac soils (*R* = 0.135, *P* < 0.001), but was hardly detectable in the bacterial communities. To evaluate the effect of straw application more specifically, the datasets were completely dissected so that samples representing one field location, one type of crop rotation and one compartment were analyzed individually per time point. This revealed a straw effect in the majority of cases (approx. 70% of all datasets) according to ANOSIM (Supplementary Table 4). More significant and higher *R*-values were observed for fungal than bacterial communities, indicating a stronger response of fungal communities to straw application (**Figure 5**). Remarkably, responses to straw were stronger in the rhizosphere than in bulk soil. This was also seen when applying ANOSIM to less dissected datasets (Supplementary Table 5). A clear trend over time concerning the responses of bacterial and fungal communities to straw application was not evident.

To identify bacterial and fungal genera that showed a significant increase in relative abundance due to straw application, we performed an analysis in STAMP. Samples with straw application were compared to those without straw application, resolved for each field location, crop rotation regime, and compartment (full list of genera in Supplementary Tables 6E,F). A specific enrichment of bacterial and fungal genera due to straw application was predominantly observed in the Italian soils and in the rhizosphere of Tarlac MR soil. Between 5 and 32 different bacterial genera and 2 to 4 fungal genera were identified per compartment (**Table 2**). More than 80% of these genera were either specifically detected in the rhizosphere or in bulk soil, but not in both compartments of a soil. Most consistently detected across compartments and field locations were members in the classes *Alphaproteobacteria*, *Actinobacteria*, and *Verrucomicrobiae*. In the fungal communities, genera belonging to the *Sordariomycetes* responded most consistently. Remarkably, several fungal taxa showed a significantly higher relative abundance in rhizosphere and bulk soil samples without straw application, including members of the *Eurotiomycetes, Microbotryomycetes, Leotiomycetes, Pezizomycetes*, and *Sordariomycetes* (Supplementary Table 6F).

**Table 2:** Number of genera significantly enriched in soils with straw application.

Phylum	Class	Italy MM		Italy RR		Tarlac MR
				
		Bulk soil	Rhizosphere	Bulk soil	Rhizosphere	Rhizosphere
*Acidobacteria*	*Acidobacteria Gp1*	1	1			
	*Acidobacteria Gp4*				1	1
	*Acidobacteria Gp6*				1	
	*Acidobacteria Gp16*					1
*Actinobacteria*	*Actinobacteria*	4		3	4	4
*Armatimonadetes*	*Armatimonadetes gp4*				1	
	*Fimbriimonadia*				1	
*Bacteroidetes*	*Cytophagia*			3		1
	*Flavobacteriia*				1	
	*Sphingobacteriia*	2		3	1	
	*Unclassified Bacteroidetes*			1		
*Candidate division WPS-1*	*WPS-1*, genera incertae sedis				1	
*Firmicutes*	*Bacilli*	1		2	1	
*Planctomycetes*	*Planctomycetia*		1	1	1	
*Proteobacteria*	*Alphaproteobacteria*	5	3	11	8	7
	*Betaproteobacteria*	1			2	1
	*Deltaproteobacteria*			3		
	*Gammaproteobacteria*	1		3	2	
*Verrucomicrobia*	*Opitutae*				1	
	*Spartobacteria*			1		
	*Verrucomicrobiae*	2		1	4	1
Total number of enriched bacterial genera		17	5	32	30	16
*Ascomycota*	*Dothideomycetes*	1				
	*Eurotiomycetes*	1			1	
	*Sordariomycetes*	2	2	1	2	
*Chytridiomycota*	*Chytridiomycetes*		1			
*Zygomycota*	*Mucoromycotina*, class incertae sedis			1		
Total number of enriched fungal genera		4	3	2	3	


## Discussion

### Relevance of Crop Rotation and Straw Application on Soil Microbial Communities in Relation to Field Location, Compartment, and Time

The effect of two agricultural management practices on soil microbial communities was evaluated, i.e., responses due to the introduction of a crop rotation regime in RR soils and short-term responses to rice straw application. We assessed the effects of these management practices in relation to the impact of field location, compartment, and over time, as these factors are known to affect microbial communities in the bulk soil and rhizosphere (Castellanos et al., 2009; Lee et al., 2011; Peiffer et al., 2013). Our data revealed that diversity as well as community composition were significantly influenced by all these factors. Overall, community composition was most different in samples from different field locations and least affected by straw application. The influence of crop rotation on soil microbial community composition was intermediate compared to the impact of the other factors.

The finding that field location had the strongest impact and that soils from Italy harbored the most distinct microbial communities is in good agreement with the fact that fields in different geographic regions and climatic zones are known to contribute to differences in microbial community composition (Neufeld and Mohn, 2005; Castellanos et al., 2009; Brockett et al., 2012; Peiffer et al., 2013). Differences may also be due to variation in soil physicochemical characteristics. Variation was observed with regard to soil type, clay content, organic carbon content, pH, and water holding capacity (Supplementary Table 1), factors that are known to influence soil microbial community composition (Brockett et al., 2012; Zhao et al., 2014, 2016; Bai et al., 2017). Besides, the experimental setup has possibly contributed to the observed differences between Italian and Philippine soils to some extent. The experiment with Italian soil was performed in a different greenhouse and with a different maize variety compared to the Philippine soils (**Table 1**). To assess the possible impact related to this difference, a control experiment with RR soil from IRRI was included in the microcosm experiment that was performed with the Italian soils. It revealed that the methodological difference was not the major factor for the observed differences between Italian and Philippine soils, because this IRRI soil remained clearly different from the Italian soils and similar, though not identical, to the other IRRI soils (Supplementary Figure 5).

Besides field location, compartment contributed substantially to the overall differences in soil microbial community composition and diversity, though the impact was weaker compared to field location (**Figure 3**), likewise as observed in previous studies (Castellanos et al., 2009; Peiffer et al., 2013). Moreover, differences in community composition between rhizosphere and bulk soil were smaller for fungal communities than for bacterial communities, as reported earlier (Pausch et al., 2016; Uroz et al., 2016; Granzow et al., 2017). The factor time also caused variation in fungal and bacterial community composition and diversity (**Figure 3** and Supplementary Table 2). The variation over time was lower in bulk soil than in the rhizosphere (Supplementary Table 5). This was expected, as microbial communities are known to develop in the rhizosphere over time along with the plant (Smalla et al., 2001; Wang et al., 2016; Qiao et al., 2017). Changes in the bulk soil can be explained by the straw degradation process, which induces successional changes in microbial community composition during residue decomposition (Bastian et al., 2009; Conrad et al., 2012; Tardy et al., 2015). This process may also have contributed to the time-dependent changes observed in the rhizosphere.

### Impact of Crop Rotation on Microbial Community Composition and Diversity

The effect of crop rotation on microbial community composition and diversity was evident in soils from all three field locations, but strongest in the Italian soils, where rice and maize were cultivated in monosuccession in adjacently located fields for more than 30 years (**Figure 3** and Supplementary Table 2). In the Philippine soils, where the impact of MR crop rotation was evaluated in comparison to RR, changes in the crop rotation regime occurred for shorter periods of time, so that microbial communities may not yet have fully adapted to the altered conditions. Nevertheless, effects of crop rotation on microbial community composition were evident (**Figure 3**). Responses to crop rotation were reported in previous studies, in which the soil bacterial and fungal community composition was analyzed in paddy soils under different crop rotation regimes such as winter wheat–rice or alfalfa–rice (Lopes et al., 2014; Zhao et al., 2014). Moreover, our findings are in agreement with Breidenbach et al. (2015), who showed that the introduction of a MR crop rotation practice does not change the structure of the bacterial community drastically within the first 3 years after introducing a MR crop rotation regime.

Two major factors may have contributed to the crop rotation dependent differences, the regular flooding of the fields, leading to periodically anoxic conditions, and the influence of crops that were repeatedly cultured in the soil. Host plant specific rhizosphere communities are known to develop due to plant species specific rhizodeposition processes (Berg and Smalla, 2009; Ladygina and Hedlund, 2010). The long-term release of plant species specific carbon compounds under maize versus rice monosuccession has probably supported the enrichment of a specific microbial community in soil. Moreover, the soil microbiota may have been affected by crop rotation specific management practices such as fertilization regimes, plant residue input, or pest control treatments (Dick, 1992; Hussain et al., 2009; Thiele-Bruhn et al., 2012; Geisseler and Scow, 2014). In particular the regular change between oxic and anoxic conditions in RR and MR soils vs. MM soils, which limits oxygen availability in soil, has to be considered, because oxygen availability is a well-known factor shaping microbial community composition (Noll et al., 2005; Kikuchi et al., 2007; Zhao et al., 2014). The relevance of this factor is confirmed by the finding that diverse facultative and obligate anaerobic microorganisms were enriched in all RR soils and were not in all cases strongly depleted in MR soils (**Figure 4**). When comparing the differences in microbial community composition between soils under different crop rotation regimes, it is obvious that MR soils are still largely similar to RR soils, while MM soils are clearly distinct from the corresponding RR soil (**Figure 1**). The introduction of flooding periods in an upland soil has obviously a much stronger impact on the microbial community composition than the extension or reduction of recurring flooding periods, as it occurring in RR and MR soils. Other studies also suggest that periodically anoxic conditions in MR crop rotation systems help to maintain a community structure similar to those in RR soils (Zhao et al., 2014; Breidenbach and Conrad, 2015).

### Microbial Taxa Responding to Crop Rotation

In response to crop rotation, the highest numbers of bacterial and fungal genera were identified in the Italian soils, which is in agreement with the strong differences observed in NMDS plots and ANOSIM analyses for these soils (**Figure 3**). In the RR soils, diverse facultative and obligate anaerobic bacterial genera were significantly enriched (**Figure 4** and Supplementary Tables 6A,B), many of them well-known as colonizers in rice field soils (Lu et al., 2006; Ahn et al., 2012; Knief et al., 2012; Itoh et al., 2013; Lopes et al., 2014; Edwards et al., 2015). These include members of the phylum *Verrucomicrobia* (*Prosthecobacter* and *Opitutus*) as well as members of the classes *Anaerolineae, Ignavibacteria*, *Negativicutes*, and *Clostridia*. Moreover, several genera of deltaproteobacterial sulfate and iron reducers (*Geobacter* and *Anaeromyxobacter*), methanotrophic bacteria (*Methylocaldum* and *Methylomonas*), and methanogenic archaea (*Methanobacterium* and *Methanomassiliicoccus*) were specifically enriched in RR soils.

In MM and MR soils, several genera belonging to the classes *Alphaproteobacteria*, *Gammaproteobacteria*, *Bacilli*, and *Actinobacteria* were specifically enriched (**Figure 4**). These are commonly detected in soil, including maize field soils (Li et al., 2014; Zhao et al., 2016; Benitez et al., 2017) or crop rotation systems with maize (Zhao et al., 2014). Interestingly, the nitrifying bacterial genera *Nitrospira* and *Nitrosococcus* were enriched in the Italian RR soil, while the archaeal genus *Nitrososphaera* was enriched in the corresponding MM soil. Thus, a switch from bacterial to archaeal nitrification appears to be linked to RR versus MM monosuccession. Similarly, Breidenbach et al. (2015) observed that members of the genus *Nitrososphaera* were enriched in a MR soil compared to RR soil. However, in some other studies, ammonium oxidizing archaea were found to be more abundant and active under the oxygen-limiting conditions in rice field soils (Wang et al., 2014, 2015, 2017; Ke et al., 2015; Azziz et al., 2016).

The number of fungal genera that were influenced by crop rotation was lower, which corresponds to the lower richness in the fungal communities. Among the fungi enriched in the Italian MM soil were *Leotiomycetes* and *Glomeromycetes*. Members of *Leotiomycetes* are known as maize endophytes (Wang et al., 2006) and *Glomeromycetes* are well-known as arbuscular mycorrhizal symbionts of maize, including the enriched genus *Entrophospora* (Na Bhadalung et al., 2005; Colombo et al., 2017). Actually, the genus *Entrophospora* was enriched in the MM bulk soil rather than in the rhizosphere (Supplementary Table 6B). This fungus obviously did not undergo a symbiotic interaction with maize in our microcosm experiment, possibly because we applied fertilizer to provide sufficient nutrients for plant growth (Na Bhadalung et al., 2005). Moreover, *Piriformospora* was significantly enriched in MM bulk soil and the RR rhizosphere soil. The specific enrichment in RR rhizosphere soil rather than in the maize rhizosphere is surprising, as this fungus is better known for its association with maize (Qiang et al., 2012). Further genera known as plant endophytes were enriched in RR or MM soil, including *Pyrenochaetopsis, Exophiala, Penicillium, Paecilomyces*, and *Preussia* (Bilal et al., 2017; Papizadeh et al., 2017). Besides the enrichment of beneficial fungi, a maize pathogen, *Ustilago*, was found in the maize rhizosphere in MM soil (Brefort et al., 2009). Taken together, these findings demonstrate very well that crop monosuccession regimes lead to the enrichment of host-plant specific beneficial as well as pathogenic microorganisms.

Members of the *Dothideomycetes* and *Chytridiomycetes* were more specifically detected in the Italian RR soil than in MM soil. The *Dothideomycetes* are a diverse class of fungi, including saprobic and aquatic organisms (Hyde et al., 2013). Similarly, the RR enriched genera *Delfinachytrium, Aquamyces, Betamyces*, and *Udeniomyces*, representing *Chytridiomycetes* and *Tremellomycetes*, are usually known from aquatic habitats (Brizzio et al., 2007; Letcher et al., 2008; Vélez et al., 2013; Zhang et al., 2016). These genera are obviously capable to establish populations in paddy rice ecosystems. Several further genera that were significantly enriched in RR or MM soils and are involved in the degradation of organic material, e.g., *Thermomyces*, *Chaetosphaeria, Mrakia*, or *Udeniomyeces* (Reblova and Winka, 2000; Brizzio et al., 2007; Zhang et al., 2015). In conclusion, these findings suggest that the specific enrichment of fungal taxa in MM or RR soils is partly driven by the flooding regime during rice cultivation, leading to the enrichment of fungi that are known from aquatic environments, as well as by the plant, leading to the enrichment of plant-host specific symbionts and pathogens. Moreover, saprotrophic fungi are affected, probably by the supply of organic carbon compounds, which differ to some extent in dependence on the cultivated crop.

### Compartment-Specific Responses to Crop Rotation

Differences in response to crop rotation were not only evident in the bulk soil, but seen to a similar extent in the maize rhizosphere according to ANOSIM results. Even over time, i.e., up to 43 or 85 days of plant development, the maize rhizosphere microbiota remained clearly distinct in the soils under different crop rotation regimes (Supplementary Table 3). This demonstrates that crop rotation regimes do not only affect bulk soil microbial communities, but also those in the plant rhizosphere. The compartment-specific analysis in STAMP revealed that more genera responding to crop rotation were identified in the bulk soil than in the rhizosphere (Supplementary Figure 3). However, the rhizosphere microbiota is less diverse compared to bulk soil (Peiffer et al., 2013), resulting in a lower number of potentially responsive taxa. A very clear response of the rhizosphere microbiota was not necessarily expected, because these microorganisms are largely controlled by plant root released carbon, which is known to shape the rhizosphere microbiota (Berg and Smalla, 2009; Bulgarelli et al., 2013). The high overlap of responsive genera in the bulk soil and rhizosphere (Supplementary Figure 3) indicates that part of the rhizosphere response is identical to that in bulk soil. This may to some extent be attributed to bulk soil organisms residing in the rhizosphere. Bacterial taxa occurring in bulk soil probably inhabit to some extent the rhizosphere without being part of a very specific plant-supported rhizosphere microbiota, especially when considering that the transition from rhizosphere to bulk soil is continuous. With increasing distance from the plant root surface, the number of plant-supported microorganisms will gradually decrease and bulk soil microorganisms will increase in relative abundance.

### Impact of Straw Application on Microbial Community Composition and Diversity in Bulk Soil and Rhizosphere

The short-term responses to straw application were rather weak compared to the other factors in soils from all three field locations (**Figures 1**, **3**). They became evident only after excluding the variation caused by field location, compartment, crop rotation (Supplementary Table 5) and time (Supplementary Table 4). In contrast to our results, Tardy et al. (2015) observed that straw application had a stronger impact than crop rotation when comparing grassland with cropland soil. The higher impact of crop rotation in our study can in case of the Italian soils be explained by the highly different cropping conditions for rice versus maize, which induced substantial changes in the microbial communities. At the Philippine sites, the strength of the impact of crop rotation and straw application was roughly equal, at least for the fungal communities, largely due to the lower impact of crop rotation at these sites (**Figure 3**). After the complete dissection of the datasets, the response of the microbial community to straw application became more evident (Supplementary Table 4).

Straw application affected the fungal communities more strongly than the bacterial communities (**Figure 5** and Supplementary Table 5). Saprotrophic fungi are known as effective decomposers contributing to the decomposition of organic matter and thus boost carbon mineralization in soil (Kjøller and Struwe, 2002; Crowther et al., 2012; Dini-Andreote et al., 2016). Moreover, fungi have been reported to be the dominant group involved in rice straw degradation in RR soils under oxic conditions (Nakamura et al., 2003). We observed a straw-dependent enrichment of genera in the classes *Sordariomycetes* and *Dothideomycetes* (family *Sporormiaceae*) in the Italian soils. These are known to play a role in the degradation of plant residue (Zhao et al., 2013; Tardy et al., 2015; Phukhamsakda et al., 2016). Remarkably, the number of fungal genera that were identified as significantly enriched was higher in treatments without straw than in treatments with straw (Supplementary Table 6F). This was observed in the rhizosphere as well as in bulk soil. Selective grazing may have affected the fungal populations in straw-supplemented soils due to the presence of high amounts of organic substrate and thus higher overall biological activity, possibly leading to a decrease in relative abundance of fungal taxa in the straw-supplemented soils. To elucidate this phenomenon in more detail, absolute abundances of selected taxa would have to be evaluated and ^13^C-straw labeling experiments could be performed to study the flow of carbon into the microbial food web in more detail.

In the bacterial community, straw application resulted in an enrichment of diverse bacterial genera (**Table 2**). Several of them are known as straw or plant residue degrading organisms, including members of *Verrucomicrobiae, Actinobacteria, Bacteroidetes* or the different classes of *Proteobacteria* (Bernard et al., 2007; Semenov et al., 2012; Pascault et al., 2013; Fan et al., 2014). The bacterial genera that were enriched by straw in the rhizosphere belong to phyla and classes that are well known to colonize the (maize) rhizosphere (Bulgarelli et al., 2013; Da Rocha et al., 2013; Peiffer et al., 2013; Hernández et al., 2015). Most genera were detected in either the rhizosphere or the bulk soil of a soil, but not in both compartments. This compartment specific response indicates that rhizosphere-inhabiting microorganisms may profit from plant-derived carbon as well as carbon available from straw application. This was proposed earlier by Shrestha et al. (2011), who studied the assimilation of rice straw in the rhizosphere of rice plants.

Analysis of similarity results suggested that the application of straw can induce stronger changes in the rhizosphere microbial communities than in bulk soil communities (**Figure 5** and Supplementary Table 5). STAMP analysis confirmed this in case of the Tarlac MR soil by identifying a higher number of genera being significantly enriched in the rhizosphere (**Table 2**). A stronger response to straw in the rhizosphere would not necessarily be expected, as the rhizosphere microbiota is considered to be mainly influenced by the plant (Berg and Smalla, 2009; Bulgarelli et al., 2013). However, the availability of easy to degrade plant root exudates may have stimulated rice straw degradation in the rhizosphere. These processes may have resulted in stronger shifts in the microbial community composition upon straw application in the rhizosphere. Such rhizosphere priming effects are well known and can improve the plant nutrient status by releasing nutrients upon mineralization of more difficult to degrade organic carbon compounds (Huo et al., 2017).

## Conclusion

Field location, followed by crop rotation, incubation time, and compartment were identified as main factors influencing microbial community composition and diversity, while the addition of straw had a minor effect. The analysis of the Italian soils revealed that long-term monosuccessionally managed soils developed substantial differences in microbial community composition, which could be well explained by alterations in oxygen availability in soil and the different cultivated crops, leading to the enrichment of plant species-specific microbial mutualists as well as pathogens. Moreover, the plant species-specific carbon supply into the soil most likely influenced the heterotrophic soil microbial community. Soils under MR crop rotation harbored microbial communities that were more alike those in rice soil than in maize soil. Obviously, the anaerobic microbial population is largely maintained in MR soils. Nevertheless, differences between RR and MR were mostly due to a depletion of anaerobic microorganisms in the MR soils. This is in agreement with the expectation that the ecologically more friendly MR crop rotation practice in comparison to RR leads to a reduction of greenhouse gas emissions (Weller et al., 2015). The short-term responses to the addition of straw became most evident after exclusion of all other factors assessed in this study. The fungal community responded more strongly than the bacterial community, but in contrast to the bacterial community more taxa of the fungal community were depleted in relative abundance in the presence of straw than enriched. It will be of interest to assess in the future also the longer-term responses of the soil and rhizosphere microbiota to recurring straw applications and microbial carbon cycling in such agricultural systems. Both management practices, crop rotation and straw application, affected not only the microbial community in the bulk soil, but to roughly similar extent those in the rhizosphere. This indicates that the rhizosphere microbiota is influenced by crop rotation and may not only profit from root-derived carbon. Actually, this influence may increase with decreasing distance to the root, as the transition from the rhizosphere to bulk soil is continuous.

## Author Contributions

SM, KF, and CK conceived and designed the research, and wrote the paper. SM performed the experiments. SM, ML, and CK analyzed the data. All authors discussed the results and approved the paper.

## Conflict of Interest Statement

The authors declare that the research was conducted in the absence of any commercial or financial relationships that could be construed as a potential conflict of interest.
