# Supplementary material for: Crop Rotation and Straw Application Impact Microbial Communities in Italian and Philippine Soils and the Rhizosphere of Zea mays
Source: Front Microbiol. 2018 Jun 15;9:1295. doi: 10.3389/fmicb.2018.01295 (PMC6013709; doi:10.3389/fmicb.2018.01295)
Supplement: Supplementary file 1 [file Data_Sheet_1.pdf]

Supplementary Material

Crop rotation and straw application impact microbial communities in Italian and Philippine soils and the rhizosphere of *Zea mays*

Sarah Maarastawi, Katharina Frindte, Marius Linnartz, Claudia Knief*

*** Correspondence:** Corresponding Author: knief@uni-bonn.de

1 Supplementary Figures and Tables

1.1 Supplementary Figures

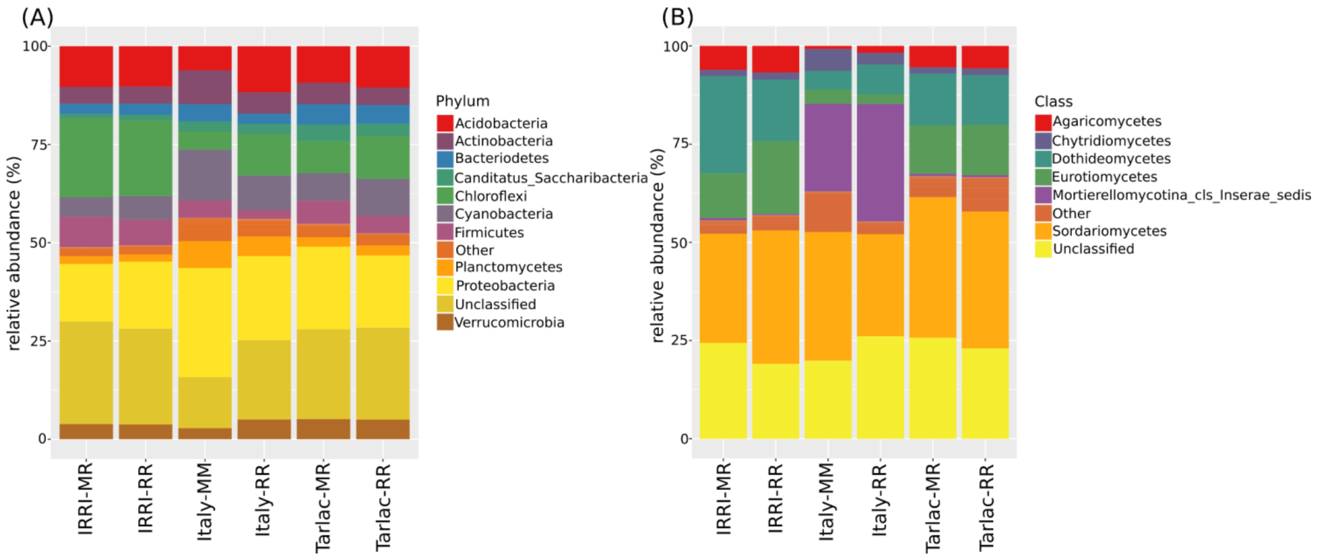

Supplementary Figure 1: Stacked bar diagrams showing the relative abundance of bacterial phyla (A) and fungal classes (B) in samples from fields undergoing different crop rotation regimes. Phyla and classes with < 2 % relative abundance are grouped and displayed as "Other".

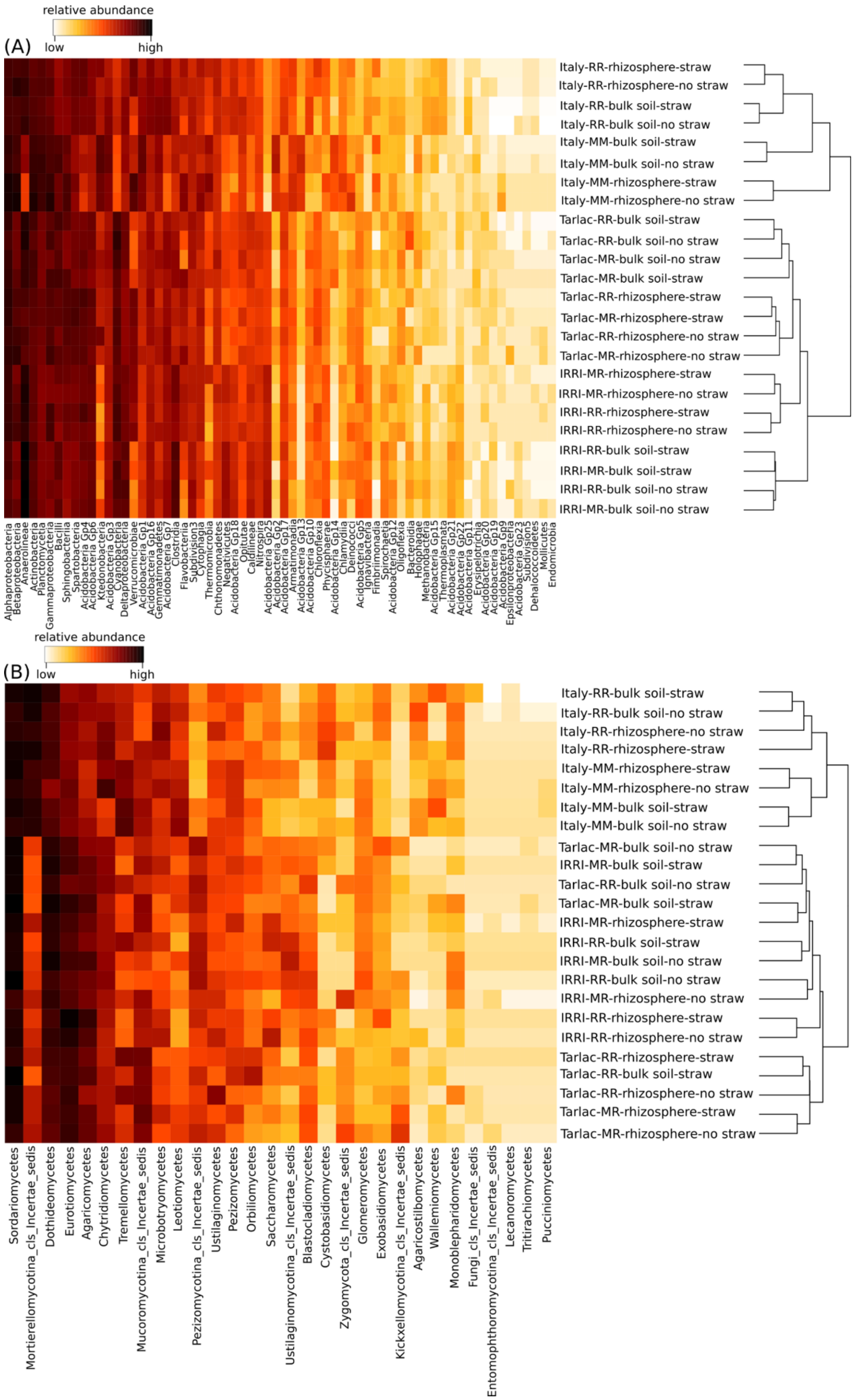
relative abundance
(A)
low
high
Italy-RR-rhizosphere-straw
Italy-RR-rhizosphere-no straw
Italy-RR-bulk soil-straw
Italy-RR-bulk soil-no straw
Italy-MM-bulk soil-straw
Italy-MM-bulk soil-no straw
Italy-MM-rhizosphere-straw
Italy-MM-rhizosphere-no straw
Tarlac-RR-bulk soil-straw
Tarlac-RR-bulk soil-no straw
Tarlac-MR-bulk soil-no straw
Tarlac-MR-bulk soil-straw
Tarlac-RR-rhizosphere-straw
Tarlac-MR-rhizosphere-straw
Tarlac-RR-rhizosphere-no straw
Tarlac-MR-rhizosphere-no straw
IRRI-MR-rhizosphere-straw
IRRI-MR-rhizosphere-no straw
IRRI-RR-rhizosphere-straw
IRRI-RR-rhizosphere-no straw
IRRI-RR-bulk soil-straw
IRRI-MR-bulk soil-straw
IRRI-RR-bulk soil-no straw
IRRI-MR-bulk soil-no straw
Alphaproteobacteria
Betaproteobacteria
Anaerolineae
Actinobacteria
Planctomycetia
Gammaproteobacteria
Bacilli
Sphingobacteriia
Spartobacteria
Acidobacteria Gp4
Acidobacteria Gp6
Ktedonobacteria
Acidobacteria Gp3
Cyanobacteria
Deltaproteobacteria
Verrucomicrobiae
Acidobacteria Gp1
Acidobacteria Gp16
Gemmatimonadetes
Acidobacteria Gp7
Clostridia
Flavobacteriia
Subdivision3
Cytophagia
Thermomicrobia
Chthonomonadetes
Negativicutes
Acidobacteria Gp18
Opitutae
Caldilineae
Nitrospira
Acidobacteria Gp25
Acidobacteria Gp2
Acidobacteria Gp17
Armatimonadia
Acidobacteria Gp13
Acidobacteria Gp10
Chloroflexia
Phycisphaerae
Acidobacteria Gp14
Chlamydiia
Deinococci
Acidobacteria Gp5
Ignavibacteria
Fimbriimonadia
Spirochaetia
Acidobacteria Gp12
Oligoflexia
Bacteroidia
Holophagae
Methanobacteria
Acidobacteria Gp15
Thermoplasmata
Acidobacteria Gp21
Acidobacteria Gp22
Acidobacteria Gp11
Erysipelotrichia
Acidobacteria Gp20
Acidobacteria Gp19
Acidobacteria Gp9
Epsilonproteobacteria
Acidobacteria Gp23
Subdivision5
Dehalococcoidetes
Mollicutes
Endomicrobia
relative abundance
(B)
low
high
Italy-RR-bulk soil-straw
Italy-RR-bulk soil-no straw
Italy-RR-rhizosphere-no straw
Italy-RR-rhizosphere-straw
Italy-MM-rhizosphere-straw
Italy-MM-rhizosphere-no straw
Italy-MM-bulk soil-straw
Italy-MM-bulk soil-no straw
Tarlac-MR-bulk soil-no straw
IRRI-MR-bulk soil-straw
Tarlac-RR-bulk soil-no straw
Tarlac-MR-bulk soil-straw
IRRI-MR-rhizosphere-straw
IRRI-RR-bulk soil-straw
IRRI-MR-bulk soil-no straw
IRRI-RR-bulk soil-no straw
IRRI-MR-rhizosphere-no straw
IRRI-RR-rhizosphere-straw
IRRI-RR-rhizosphere-no straw
Tarlac-RR-rhizosphere-straw
Tarlac-RR-bulk soil-straw
Tarlac-RR-rhizosphere-no straw
Tarlac-MR-rhizosphere-straw
Tarlac-MR-rhizosphere-no straw
Sordariomycetes
Mortierellomycotina_cls_Incertae_sedis
Dothideomycetes
Eurotiomycetes
Agaricomycetes
Chytridiomycetes
Tremellomycetes
Mucoromycotina_cls_Incertae_sedis
Microbotryomycetes
Leotiomycetes
Pezizomycotina_cls_Incertae_sedis
Ustilaginomycetes
Pezizomycetes
Orbiliomycetes
Saccharomycetes
Ustilaginomycotina_cls_Incertae_sedis
Blastocladiomycetes
Cystobasidiomycetes
Zygomycota_cls_Incertae_sedis
Glomeromycetes
Exobasidiomycetes
Kickxellomycotina_cls_Incertae_sedis
Agaricostilbomycetes
Wallemiomycetes
Monoblepharidomycetes
Fungi_cls_Incertae_sedis
Entomophthoromycotina_cls_Incertae_sedis
Lecanoromycetes
Tritirachiomycetes
Pucciniomycetes

Supplementary figure 2: Sample clustering of bacterial (A) and fungal (B) communities according to the UPGMA algorithm based on Bray-Curtis dissimilarities between groups of samples. The heat maps show *log(x+1)* transformed relative abundances of the bacterial and fungal classes, sorted by decreasing relative abundance. For the heat map, the OTUs were grouped at class level. Samples representing different time points were grouped and relative abundances were calculated based on summarized read numbers. Unclassified OTUs were excluded from the analysis. The heatmap was constructed in R using the package Heatmap3.

The clustering of groups of samples reveals that bacterial and fungal communities were well separated according to field location, and the most distinct samples were those from the Italian field sites. A clear separation according to field location was also observed for the bacterial communities in the soils from the two different Philippine sites, but this separation was weaker for the fungal communities. The impact of crop rotation was of particular strength in the Italian soils. The differences between compartments are evident, especially in the Italian soils.

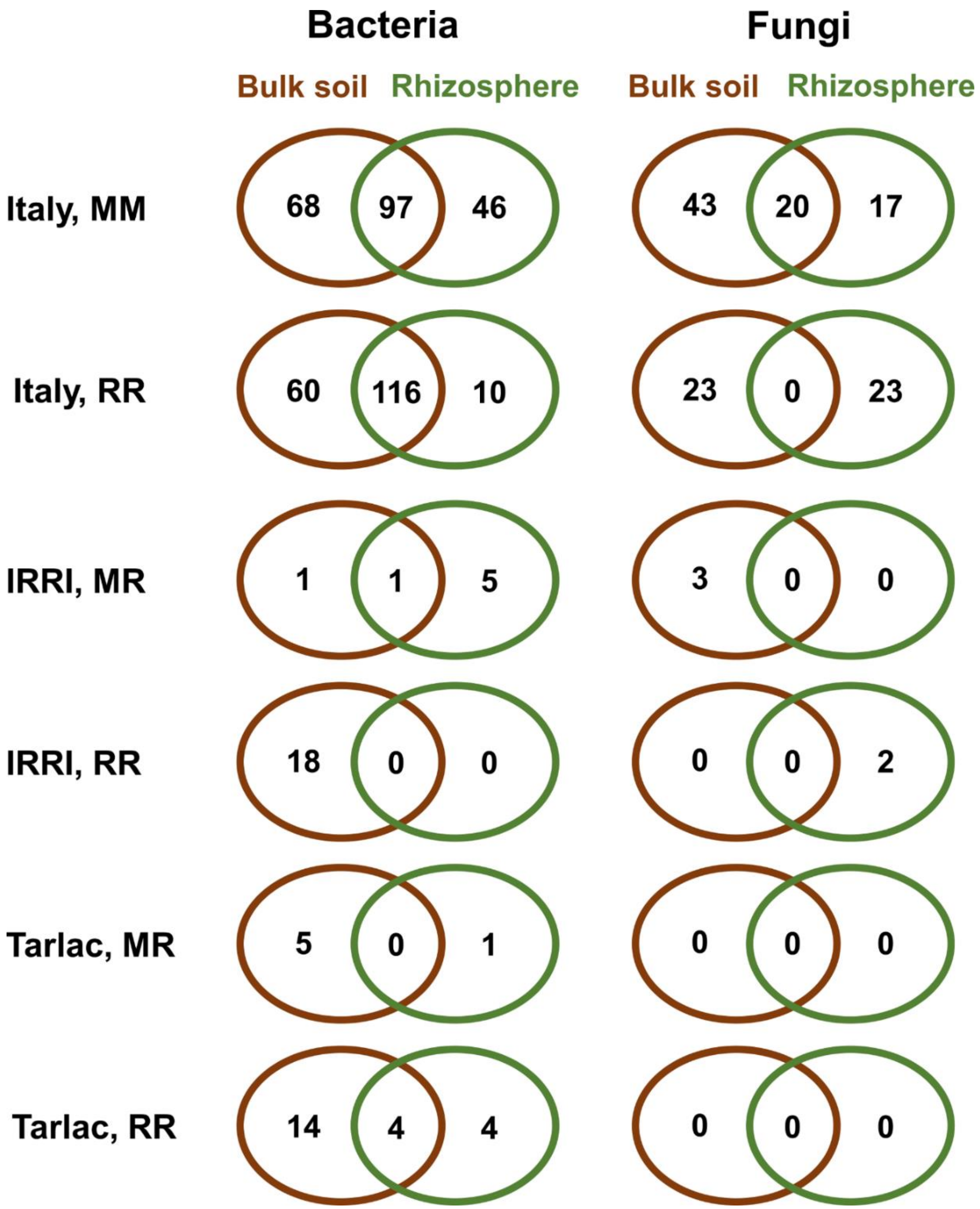

Supplementary Figure 3: Venn diagrams displaying numbers of compartment specific and compartment-independently enriched bacterial and fungal genera in dependence on crop rotation. The impact of crop rotation was analyzed in MM versus RR (Italy) or MR versus RR soils (IRRI and Tarlac) using the STAMP algorithm (list of genera in supplementary tables 6 C, D).

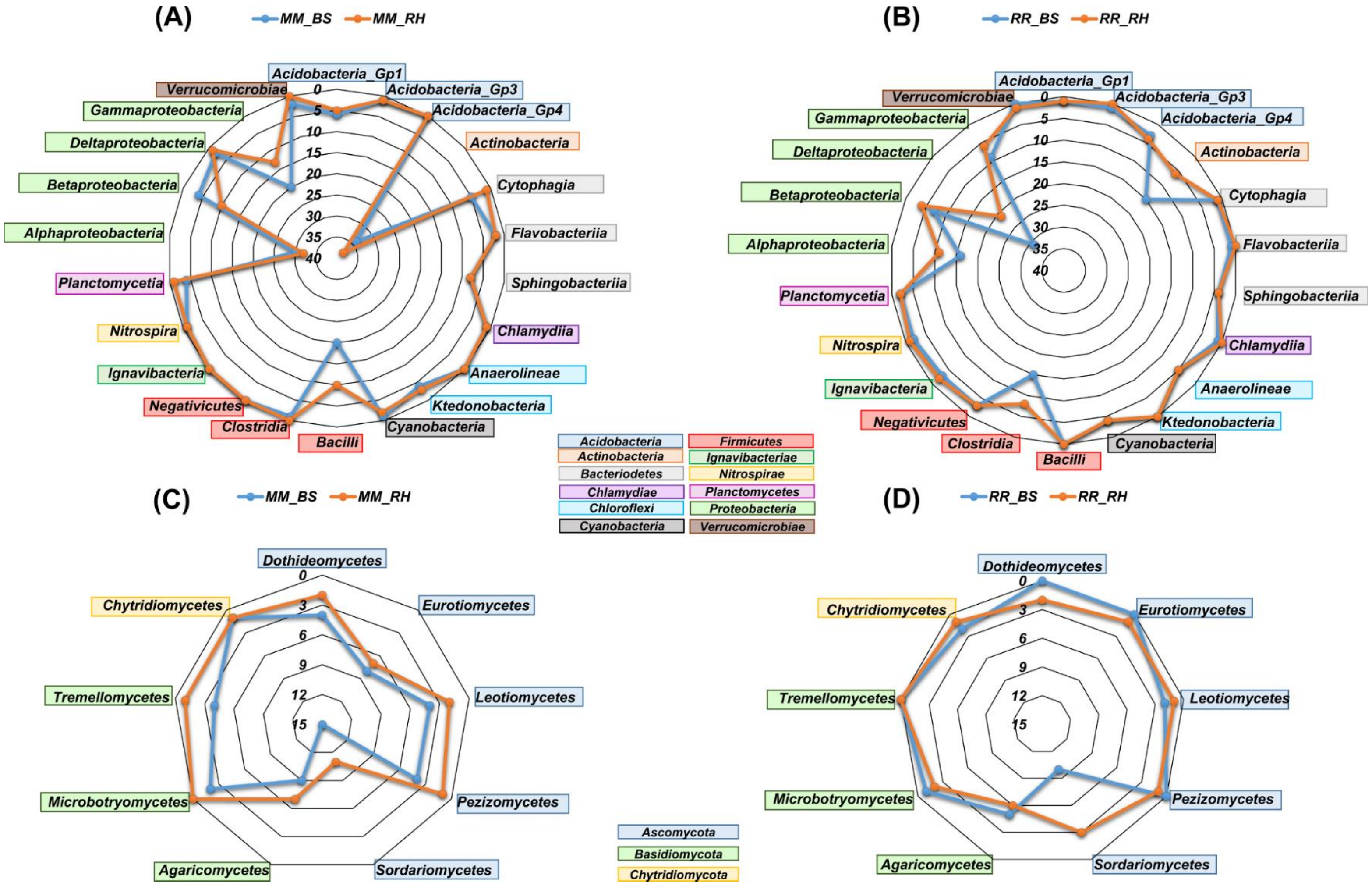

Supplementary Figure 4: Radar charts showing the number of bacterial (A), (B) and fungal (C), (D) genera in the different classes that were identified as significantly enriched by crop rotation in the different compartments (BS = bulk soil, RH = rhizosphere) based on STAMP analysis. Plots are shown for Italian MM soil (A), (C) and RR soil (B), (C). Displayed are classes for which at least three different genera were identified as specifically enriched in one or the other soil.

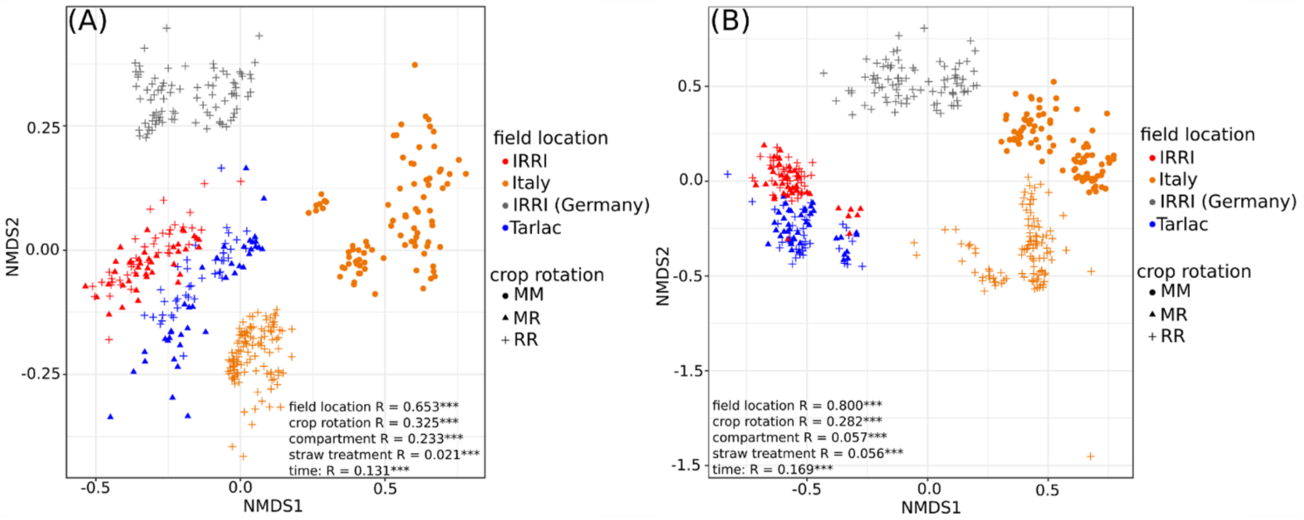

Supplementary Figure 5: Ordination plots showing the influence of field location, crop rotation, compartment, straw treatment and time on bacterial (A) and fungal (B) community composition. NMDS plots based on Bray-Curtis similarities were calculated based on relative OTU abundance. Results of ANOSIM are shown with $P < 0.05$*, $P < 0.01$**, $P < 0.001$*** for all grouping factors.

In comparison to figure 1, these plots include results obtained from "IRRI (Germany)" samples, which were collected earlier at the IRRI site from RR soils, shipped to Germany and included in the microcosm experiment performed with soils from Italy. This was done to evaluate the potential impact of the experimental study site location and the maize cultivar. The plots reveal that IRRI (Germany) samples cluster distinctly but still closely to those from IRRI, especially in case of the bacterial community. This demonstrates that we cannot exclude that the conductance of the microcosm experiments at two different locations has contributed to some extent to the observed differences between the Italian and Philippine field sites. However, the still very distinct clustering of IRRI (Germany) samples from Italy samples demonstrates that other site-specific factors contributed more substantially to the site-specific differences. A major effect of the maize cultivar can be excluded, as the rhizosphere samples clustered in all cases very closely together with the corresponding bulk soil samples in these plots.

1.2 Supplementary Tables

Supplementary Table 1: Soil parameters of homogenized soil samples, analyzed before the start of the experiment. Measurements of N_{min}, C/N and clay fraction were performed in duplicates. Mean values ± standard error are shown.

	**pH**	**N_{min} (mg/kg)**	**N (%)**	**C (%)**	**C:N**	**C_{org} (%)**	**Soil type**	**Clay fraction (%)**	**Water holding capacity (%)**
**Italy RR**	4.9	24.97 ± 1.79	0.07	0.98	12.71 ± 0.24	0.98	loam	9.51 ±0.47	41.8
**Italy MM**	4.2	27.63 ± 0.46	0.06	0.74	11.11 ± 0.89	0.75	sandy loam	13.22 ±0.34	43.9
**IRRI RR**	5.7	6.81 ± 0.14	0.14	1.73	11.89 ± 0.51	1.74	silty clay	59.56 ±0.35	79.9
**IRRI MR**	5.7	4.71 ± 0.05	0.15	1.81	11.94 ± 0.86	1.82	silty clay	60.17 ± 0.04	72.7
**Tarlac RR**	5.8	3.89 ± 0.02	0.06	0.77	11.67 ± 0.22	0.77	loam	10.19 ± 0.08	60.3
**Tarlac MR**	5.2	4.40 ± 0.09	0.06	0.95	13.96 ± 0.01	0.96	silty loam	12.87 ± 0.09	53.9

Supplementary Table 2: Richness and diversity of fungal and bacterial communities. Numbers represent mean values ± standard deviation.

		**Bacteria**				**Fungi**			
**Treatments**		**Richness**	***P*-value**	**Chao1 diversity**	***P*-value**	**Richness**	***P*-value**	**Chao1 diversity**	***P*-value**
**Field location**	Italy	1221 ± 253	< 0.001	1974 ± 253	< 0.001	176 ± 49		316 ± 113	< 0.001
	IRRI	1347 ± 135		1984 ± 255		198 ± 45		314 ± 76	
	Tarlac	1523 ± 201		2386 ± 389		214 ± 53		373 ± 106	
**Crop rotation**	RR	1401 ± 192	< 0.001	2221 ± 368	< 0.001	195 ± 52	< 0.001	348 ± 112	< 0.001
	MM	1022 ±209		1641 ± 431		155 ± 28		260 ± 64	
	MR	1417 ± 172		2138 ± 345		217 ± 50		357 ± 100	
**Compartment**	bulk soil	1396 ± 212	< 0.001	2188 ± 397	< 0.001	199 ± 47	< 0.001	310 ± 104	< 0.001
	rhizosphere	1207 ± 260		1903 ± 460		178 ± 55		300 ± 104	
**Straw treatment**	no straw	1297 ± 245	0.015	2035 ± 436	0.018	207 ± 51	< 0.001	356 ± 109	< 0.001
	straw	1331 ± 241		2094 ± 434		173 ± 46		301 ± 97	
**Time**	0	1519 ± 156	< 0.001	2387 ± 328	< 0.001	208 ± 50	< 0.001	372 ± 130	< 0.01
	8	1212 ± 207		1960 ± 434		175 ± 54		322 ± 123	
	15	1307 ± 202		2026 ± 376		183 ± 42		306 ± 83	
	29	1163 ± 287		1883 ± 502		166 ± 38		290 ± 80	
	43	1347 ± 275		2057 ± 487		206 ± 59		348 ± 111	
	85	1129 ± 239		1786 ± 433		154 ± 24		250 ± 52	

Supplementary Table 3: Influence of crop rotation on microbial community composition according to R-values derived from an ANOSIM. $P < 0.001$***, $P < 0.01$**, $P < 0.05$*.

	**Time**	**0**	**8**	**15**	**29**	**43**	**85**
**16S rRNA gene sequence data**	Italy bulk soil	1***	1***	1***	1***	1***	1***
	Italy rhizosphere		1***	1***	0.936***	1***	1***
	IRRI bulk soil	0.456**		0.531***		0.536***	
	IRRI rhizosphere			0.826***		0.287**	
	Tarlac bulk soil	0.442**		0.486***		0.143*	
	Tarlac rhizosphere			0.462***		0.510***	
**ITS1 sequence data**	Italy bulk soil	0.509***	0.882***	0.793***	0.865***	0.843***	0.714***
	Italy rhizosphere		0.119*	0.514***	0.950***	0.428***	0.766***
	IRRI bulk soil	0.153*		0.164*		0.174*	
	IRRI rhizosphere			0.282**		0.310**	
	Tarlac bulk soil	0.189**		0.217**		0.214**	
	Tarlac rhizosphere			0.222**		0.436***	

The calculation of mean R-values from this table and comparison by ANOVA demonstrated that the response to crop rotation was significantly stronger in Italian soils than in Philippine soils ($P < 0.001$) and that fungal communities showed a stronger response than bacterial communities (paired t-test, $P < 0.005$).

Supplementary Table 4: Influence of straw mulching on microbial community composition according to R-values derived from an ANOSIM. *P* < 0.05*.

		Time	0	8	15	29	43	85
16S rRNA gene sequence data	Bulk soil	Italy RR	0.073	0.583*	0.438*	0.198*	0.219*	0.677*
		Italy MM	0.063	0.542*	0.406*	0.260*	0.260*	0.468*
		IRRI RR	0.260*		0.031		0.960*	
		IRRI MR	0.239*		-0.125		0.510*	
		Tarlac RR	0.146		0.125		-0.010	
		Tarlac MR	0.615*		0.072		0.135	
	Rhizosphere	Italy RR		-0.021	0.844*	0.448*	0.388*	0.760*
		Italy MM		0.406*	0.635*	0.698*	0.593*	0.135
		IRRI RR			0.292*		-0.063	
		IRRI MR			-0.073		0.844*	
		Tarlac RR			0.615*		0.427*	
		Tarlac MR			1*		0.885*	
ITS1 sequence data	Bulk soil	Italy RR	0.583*	0.729*	0.854*	0.365*	0.323*	0.385*
		Italy MM	0.083	0.500*	0.395*	0.177*	0.562*	0.281
		IRRI RR	0.970*		-0.031		0.948*	
		IRRI MR	0.281*		0.521*		0.447*	
		Tarlac RR	0.896*		0.354		0.656*	
		Tarlac MR	0.875*		0.348		-0.021	
	Rhizosphere	Italy RR		0.479*	0.062	-0.166	0.479*	1*
		Italy MM		0.656*	0.604*	1*	0.875*	1*
		IRRI RR			0.646*		0.156	
		IRRI MR			0.177*		1*	
		Tarlac RR			0.218		0.083	
		Tarlac MR			0.792*		1*	

The calculation of mean R-values from this table (including only significant and thus reliable R-values) and comparison by ANOVA demonstrated that fungal communities showed a stronger response than bacterial communities (P < 0.05) and that responses were stronger in the rhizosphere than in bulk soil (P < 0.01).

Supplementary Table 5: Influence of straw treatment and time point of sampling on bacterial and fungal community composition. R-values based on ANOSIM are presented with *P* < 0.001***, *P* < 0.01**, *P* < 0.05*.

	**Bacteria**		**Fungi**	
	**Straw**	**Time**	**Straw**	**Time**
Italy RR bulk soil	0.088**	0.472***	0.304***	0.145***
Italy RR rhizosphere	0.107**	0.684***	0.330***	0.326***
Italy MM bulk soil	0.109**	0.444***	0.181***	0.045
Italy MM rhizosphere	0.051	0.691***	0.645***	0.222***
IRRI RR bulk soil	0.055	0.569***	0.191**	0.352**
IRRI RR rhizosphere	0.001	0.696**	0.046	0.119**
IRRI MR bulk soil	-0.011	0.579***	0.223**	0.188**
IRRI MR rhizosphere	0.075	0.913***	0.410**	0.312**
Tarlac RR bulk soil	0.050	0.229***	0.320**	0.249**
Tarlac RR rhizosphere	0.175*	0.605***	0.103	0.479***
Tarlac MR bulk soil	0.085	0.310***	0.374***	0.139*
Tarlac MR rhizosphere	0.528**	0.806***	0.694***	0.463***
